# Cellular crosstalk during liver regeneration: unity in diversity

**DOI:** 10.1186/s12964-022-00918-z

**Published:** 2022-08-08

**Authors:** Wenzhi Shu, Mengfan Yang, Jiayin Yang, Shengda Lin, Xuyong Wei, Xiao Xu

**Affiliations:** 1 Key Laboratory of Integrated Oncology and Intelligent Medicine of Zhejiang Province, Department of Hepatobiliary and Pancreatic Surgery, Affiliated Hangzhou First People’s Hospital, Zhejiang University School of Medicine, Hangzhou, 310006 China; 2 Institute of Organ Transplantation, Zhejiang University, Hangzhou, 310003 China; 3 Department of Liver Surgery and Liver Transplantation Center, West China Hospital of Sichuan University, Chengdu, Sichuan People’s Republic of China; 4 NHC Key Laboratory of Combined Multi-Organ Transplantation, Hangzhou, 310003 China; 5 Zhejiang Provincial Key Laboratory for Cancer Molecular Cell Biology, Life Sciences Institute, Zhejiang University, Hangzhou, 310058 Zhejiang China; 6 Westlake Laboratory of Life Sciences and Biomedicine, Hangzhou, 310024 China; 7 Program in Clinical Medicine, Zhejiang University School of Medicine, Hangzhou, People’s Republic of China

**Keywords:** Liver regeneration, Hepatocytes, Non-parenchymal cells, Liver progenitor cells, Cellular crosstalk

## Abstract

The liver is unique in its ability to regenerate from a wide range of injuries and diseases. Liver regeneration centers around hepatocyte proliferation and requires the coordinated actions of nonparenchymal cells, including biliary epithelial cells, liver sinusoidal endothelial cells, hepatic stellate cells and kupffer cells. Interactions among various hepatocyte and nonparenchymal cells populations constitute a sophisticated regulatory network that restores liver mass and function. In addition, there are two different ways of liver regeneration, self-replication of liver epithelial cells and transdifferentiation between liver epithelial cells. The interactions among cell populations and regenerative microenvironment in the two modes are distinct. Herein, we first review recent advances in the interactions between hepatocytes and surrounding cells and among nonparenchymal cells in the context of liver epithelial cell self-replication. Next, we discuss the crosstalk of several cell types in the context of liver epithelial transdifferentiation, which is also crucial for liver regeneration.

**Video abstract**

**Supplementary Information:**

The online version contains supplementary material available at 10.1186/s12964-022-00918-z.

## Background

**Fig. 1:**
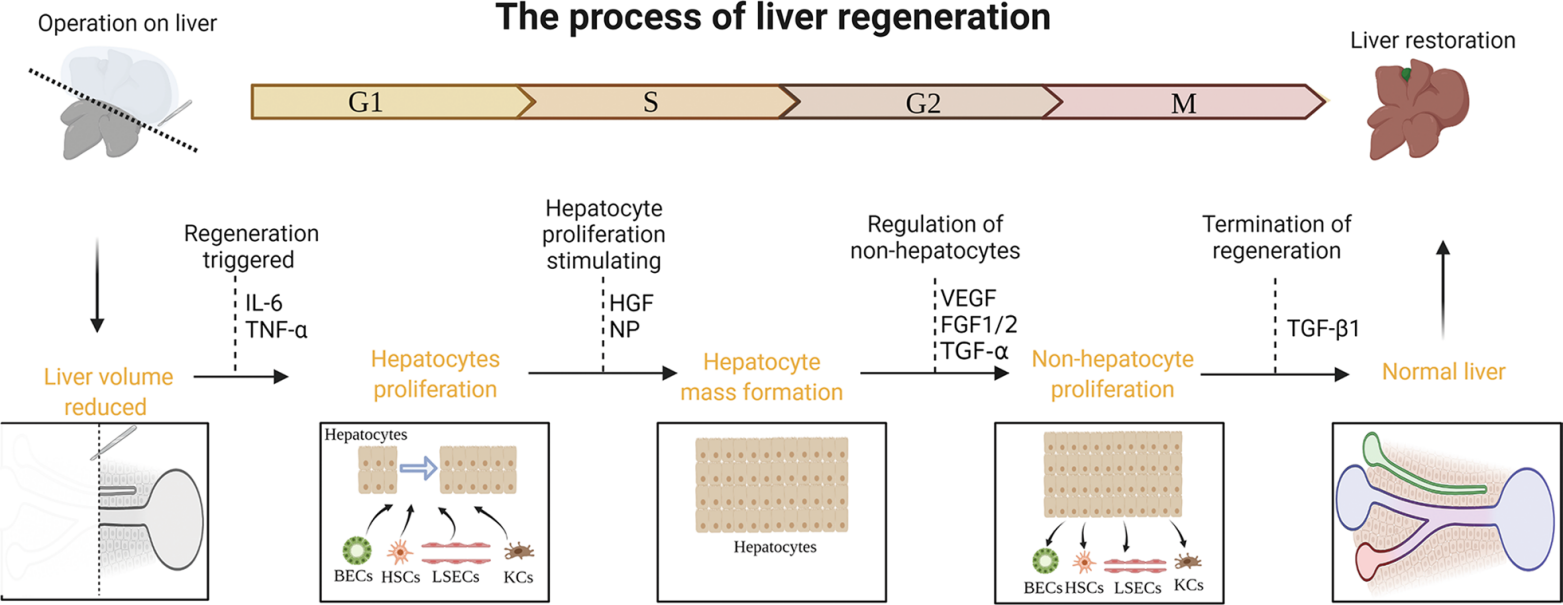
Different stages of liver regeneration. Hepatocytes are stimulated by other cells and start to proliferate; the proliferating hepatocyte clusters in turn stimulate the proliferation of other cells; the damaged liver returns to its normal structure

Although regeneration after liver injury is a continuous process, it can be artificially separated into three stages. The first stage refers to hepatocytes responding to various stimuli (from both hepatocytes and nonparenchymal cells (NPCs)) and eventually proliferating. In the second stage, replicating hepatocytes stimulate the proliferation of NPCs (such as liver sinusoidal endothelial cells (LSECs)) to adapt to the enlarged hepatocyte mass. Finally, during the termination phase, the gradual disappearance of proliferation and the induction of cell death occurs, which are critical in maintaining normal liver volume [1–3] (Fig. 1).

The liver lobule is the basic histological unit of the liver, which can be divided into three areas: around the portal vein (zone 1), around the center (zone 3), and the transition zone (zone 2) [4]. However, in recent years, gene lineage tracking methods based on Cre/LoxP technology have been used to track proliferating cells in different regions during liver regeneration more accurately and for longer than traditional technologies [5, 6]. In addition, our team’s previous research showed that a subgroup of hepatocytes with high telomerase expression have a stronger proliferation ability than other cell groups and restore liver volume when liver damage occurs [7]. Therefore, spatial heterogeneity in liver regeneration has been discovered at the cellular level; different liver cell subpopulations (both hepatocytes and NPCs) have their own contributions to regeneration under various physiological conditions.

In addition, it is generally believed that liver regeneration can be achieved through hepatocyte proliferation or hypertrophy in pathological conditions [8]. When partial hepatectomy (PHx) reaches 30%, hepatocyte hypertrophy is sufficient to restore normal liver volume. However, when the liver quality is severely damaged, such as when the PHx is as high as 70%, hypertrophy occurs, followed by cell proliferation, and the two processes restore the liver volume [8]. This finding is consistent with our previous work, where we established 1/3 and 2/3 PHx models in rats to observe the differences in miRNAs between the two groups. It was found that the changes in miRNA expression in the 2/3 PHx population were more obvious than in the 1/3 PHx population, which indicated that the livers in this group were preparing for cell proliferation [9]. These two regeneration methods have also been validated in clinical models. For example, in the associating liver partition and portal vein ligation for staged hepatectomy (ALPPS) model, not only the mitotic characteristics of hepatocytes but also extreme hypertrophy of hepatocytes and binuclear hepatocytes are observed [10]. Therefore, these studies showed that improving hypertrophy and proliferation the capacity of hepatocytes could transform the unresectable liver into resectable tissue and improve patient survival.

## Cellular crosstalk in self-replication of hepatic epithelial cells

Liver regeneration from self-replication of hepatic epithelial cells has been extensively studied, which regenerative characteristics of hepatocytes and cholangiocytes are summarized as phenotypic fidelity. As a delicately connected organ, hepatocytes cooperate precisely with surrounding cells, including biliary epithelial cells (BECs), LSECs, hepatic stellate cells (HSCs) and kupffer cells (KCs), and NPCs also closely communicate with each other to ensure this characteristic. In this section, advanced mechanisms of crosstalk between hepatocytes and surrounding NPCs are discussed in detail. At the same time, the mechanism of interaction among various nonhepatocytes cells will also be described.

### Crosstalk between hepatocytes and surrounding cells

#### Hepatocytes and LSECs: mutual precision regulation

LSECs are highly specialized endothelial cells that represent the interface between blood cells on one side and hepatocytes and HSCs on the other side [11, 12]. There are three sources of LSECs during liver regeneration: mature LSECs, intrahepatic or resident sinusoidal epithelial progenitor cells and bone marrow-derived sinusoidal epithelial progenitor cells (BM-SPCs) [13]. Experiments have shown that after 70% PHx, bone marrow-derived LSECs account for up to 25% of the total LSEC population. Studies have shown that the interaction between stromal cell-derived factor 1 (SDF-1), which is secreted by hepatocytes, and CXC chemokine receptor 7 (CXCR7), which is expressed by BM-SPCs, can promote the recruitment of BM-SPCs [14].

**Fig. 2:**
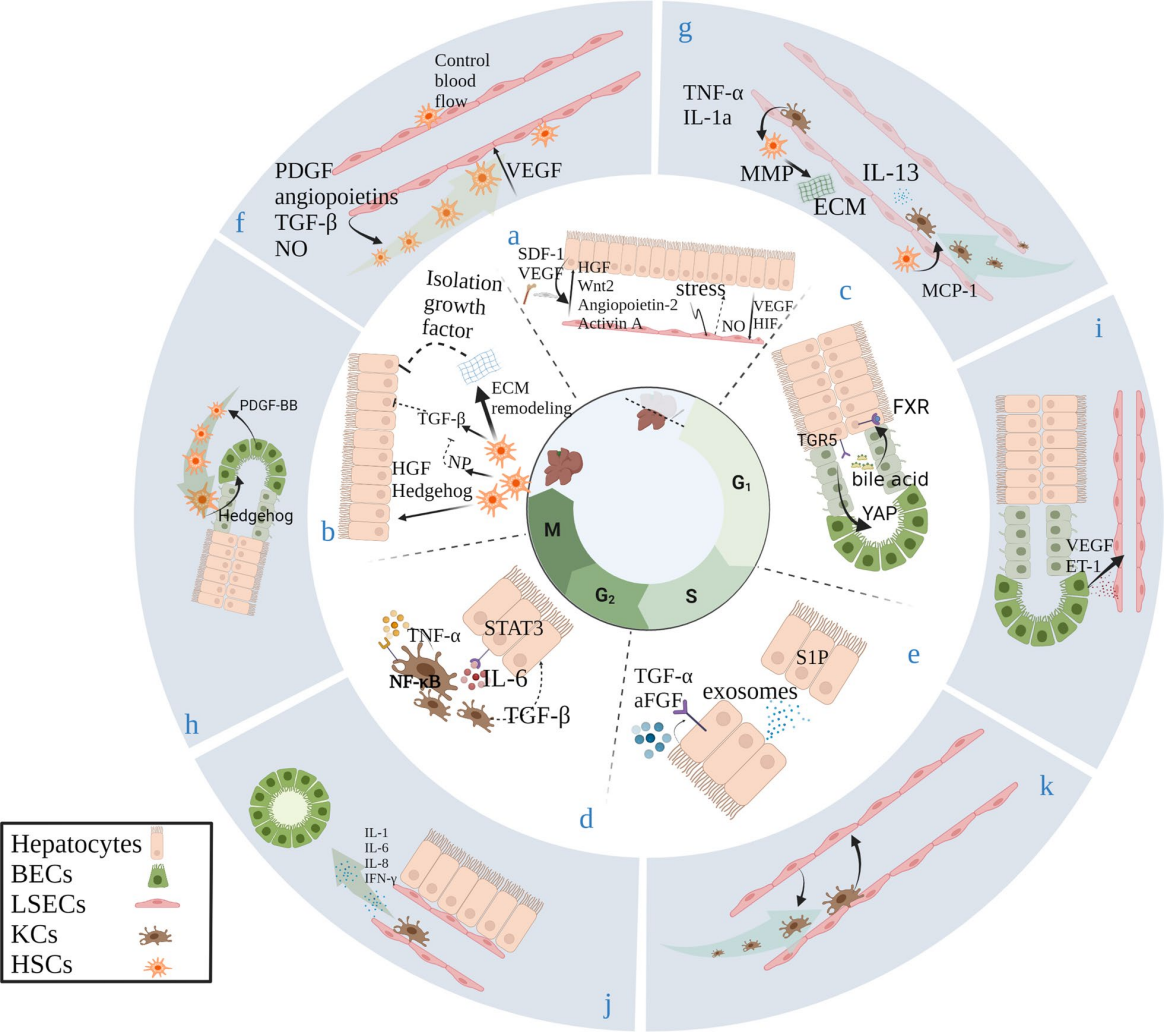
Cellular crosstalk in self-replication of hepatic epithelial cells. **a** Hepatocytes and Liver Sinusoidal Endothelial cells. **b** Hepatocytes and Hepatic Stellate cells. **c** Hepatocytes and Biliary Epithelial cells. **d** Hepatocytes and Kupffer cells. **e** Hepatocytes and Hepatocytes. **f** Liver Sinusoidal Endothelial cells and Hepatic Stellate cells. **g** Hepatic Stellate cells and Kupffer cells. **h** Hepatic Stellate cells and Biliary Epithelial cells. **i** Liver Sinusoidal Endothelial cells and Biliary Epithelial cells. **j** Kupffer cells and Biliary Epithelial cells. **k** Liver Sinusoidal Endothelial cells and Kupffer cells

Liver regeneration requires precise synchronization between hepatocytes and LSECs, and LSECs coordinate the secretion of cytokines and growth factors required for hepatocyte proliferation. In addition, LSEC proliferation is also regulated by hepatocytes [15, 16]. Liver damage increases liver vascular endothelial growth factor (VEGF) expression, promoting the recruitment of BM-SPCs rich in hepatocyte growth factor (HGF). After PHx, the portal vein flow per gram of tissue immediately increases, enhancing the shear stress on LSECs [17, 18]. Endothelial cells secrete Nitric oxide (NO) under shear stress, which promotes liver regeneration by enhancing the response of hepatocytes to HGF [19]. LSECs under shear stress also secrete Wnt protein, which regulates the increase of β-Catenin in hepatocytes and rapidly translocates to the nucleus. The increase in nuclear β-catenin regulates its target genes, such as cyclin D1, which is an important driver of liver regeneration [16, 20]. It was found that the pro-proliferation effect of LSECs on hepatocytes was regulated by the Inhibitor of DNA binding 1 (Id1) protein, which was confirmed by the decreased expression of Wnt after PHx in Id1^–/–^ mouse [13, 16]. Intracellular pathways that are activated by shear stress include the stimulation of transmembrane proteins, the activation of ion channels, the mobilization of intracellular calcium ions, the Notch1 signaling pathway, and the activation of transcription factors such as Kruppel-like factor 2 (KLF2) and vascular cellular adhesion molecule-1 (VCAM-1). Moreover, the expression of CD44, c-fos, c-myc and c-jun is involved in this process [21–24]. These molecules are crucial for the regeneration of hepatocytes. However, when PHx is excessive, shear stress damages LSECs and may cause hemorrhagic necrosis [17, 25, 26] (Fig. 2). In liver transplant recipients, "small-for-size" livers can drastically increase portal vein flow (PVF), damage the liver's regenerative capacity, and cause "small-for-size" syndrome (SFSS) [27]. Previously, our team suggested that reducing PVF through somatostatin or a mesocaval shunt (MCS) could reduce liver damage and promote regeneration [28, 29]. There is evidence that maintaining portal vein inflow to an average of 3.2 times higher than the baseline helps to promote hypertrophy in liver remnants and reduce cell apoptosis [28]. These results suggest that limiting shear stress may be a strategy to prevent liver failure due to insufficient liver regeneration after hepatectomy. HGF then stimulates the proliferation of hepatocytes to mediate liver regeneration [11]. In addition to HGF, LSECs also secrete angiogenic factors such as angiopoietin-2, fibronectin extra domain A (FEDA) and activin A [16, 30, 31]. In general, hepatocytes and LSECs are mutually activated and interdependent in liver regeneration. LSECs promote the proliferation of hepatocytes. With increasing proportions of hepatocytes, these cells subsequently encounter relative hypoxia and induce the hypoxia inducible factor (HIF) [32] pathway and downstream proangiogenic factors, which in turn promote blood vessel growth to bring more nutrients to regenerated liver cells (Fig. 1).

#### Hepatocytes and HSCs: HSCs have multiple functions

Activated HSCs (aHSCs) are located in the Disse gap between sinusoidal endothelial cells and liver epithelial cells. These cells are characterized by a long dendritic cytoplasm and the storage of vitamin A (retinol), constituting the largest vitamin A reservoir in the human body [33, 34].

aHSCs are the main source of cytokines that drive regeneration and the basic conditions for liver regeneration. One of the most critical factors produced by aHSCs is HGF, which is stored in large amounts in the extracellular matrix (ECM) and supports liver regeneration through interactions with hepatocytes [35]. Initially, HGF was identified as the mitogen of adult rat hepatocytes in the serum of 70% of rats that underwent hepatectomy [36, 37]. HGF is transported to hepatocytes through endocrine and paracrine pathways [38, 39]. Under pathological conditions, such as tissue damage, pro-HGF is converted to its biologically active form by proteolytic digestion at specific sites. This proteolytic cleavage may be mediated by urokinase plasminogen activator (u-PA) [40, 41]. HGF directly binds to the specific receptor mesenchymal–epithelial transition factor (c-MET) on the surface of hepatocytes to promote hepatocyte proliferation [42]. The binding of HGF to c-MET activates downstream signaling pathways, including the mitogen-activated protein kinase (MAPK) cascade, the PI3K-Akt axis and the nuclear factor-κb inhibitor-α (Iκbα)–nuclear factor-κb (NF-κb) complex [43–45]. MET can be linked directly and indirectly to reactive oxygen species (ROS), which activate the MARK cascade [46, 47]. Active extracellular signal-related kinases (ERKs) translocate to the nucleus, where they phosphorylate and stabilize several transcription factors involved in the early stages of the G1-S cell cycle transition. The activation of MET can also induce the translocation of STAT dimers and NF-κb to the nucleus, where they act as transcription factors to regulate the expression of several genes related to cell proliferation or differentiation [48, 49].

In addition, during the initial stage of liver regeneration, HSCs can also produce norepinephrine (NP), which is known to downregulate the inhibitory effect of transforming growth factor-beta (TGF-β) on mitosis and enhance the secretion of HGF and epidermal growth factor (EGF), affecting mitogenesis in serum-free hepatocyte culture [50–53].

In addition to these positive effects, HSCs have negative effects on liver regeneration. After the liver regenerates to the required volume in the body, the liver will produce some factors to curb DNA synthesis, and there is evidence that this effect is related to HSCs [54]. TGF-β is an effective cell proliferation inhibitor that inhibits DNA synthesis in rat hepatocytes in vitro in a dose-dependent manner. In vivo, serotonin binds to the 5-hydroxytryptamine receptor subtype 2B (5-HT2B) on the surface of HSCs to activate the expression of TGF-β1, and TGF-β1 inhibits hepatocyte proliferation through MAPK1 signaling and the transcription factor JunD [55, 56]. Moreover, during the termination phase of the regeneration response, the reconstruction of ECM by HSCs allows for the isolation of excess growth factors (HGF and FGF), prompting hepatocytes to exit the cell cycle and return to a resting state [57]. These descriptions have explained the multifaceted nature of HSCs, which are involved in the initiation and termination of regeneration, as well as ECM remodeling (Fig. 2).

#### Hepatocytes and KCs: role of the immune system

KCs are macrophages located between the sinusoidal endothelium and hepatocytes. KCs are the largest population of resident macrophages in the body, accounting for approximately 80% of the total number of macrophages [58]. Liver injury is sensed by KCs and triggers their activation, and they then release a large number of cytokines that interact with hepatocytes, promoting cell cycle transition in hepatocytes and activating a series of signaling pathways in hepatocytes to regulate liver regeneration [59–61]. During the development of liver regeneration, macrophages are highly plastic and have synergistic or opposing functions that depend on the cues they receive from the microenvironment.

The role of the immune system in liver regeneration has attracted increasing interest. KCs are considered to be the most important type of immune cells associated with liver regeneration. During the interaction between KCs and hepatocytes, the cytokines secreted by KCs, such as tumor necrosis factor-α (TNF-α) and interleukin 6 (IL-6), transform hepatocytes from the resting state to the dividing state [2, 62–64]. Once the liver is damaged, KCs may be activated by inflammatory factors such as lipopolysaccharide (LPS), C3a, C5a, and intercellular cell adhesion molecule (ICAM) and begin to produce and secrete TNF-α [65–67]. TNF-α acts in an autocrine manner and further activates NF-κB, which in turn stimulates the secretion of TNF-α and IL-6. IL-6 binds to receptors on hepatocytes, activates the STAT3 signaling pathway, and promotes hepatocyte proliferation [66]. The IL-6/STAT3 signaling pathway, which includes IL-6 receptor, gp130, receptor-associated Janus kinase (Jak) and STAT3. After gp130 recognizes IL-6, it immediately transmits IL-6 signals to hepatocytes, and gp130 is recognized by the Src homology 2 (SH2) domain of STAT3 [68]. Activated STAT3 forms a homodimer. The STAT3 dimer then translocates to the nucleus and regulates the expression of its target genes [69]. STAT3 regulates the G1/S transition of hepatocytes by controlling cyclin D1 expression. The target genes of STAT3 include FLICE inhibitory protein (FLIP), B-cell lymphoma-2 (Bcl-2) and Bcl-xL; therefore, STAT3 may have antiapoptotic effects [70].

In contrast, some studies have suggested that KCs have an inhibitory effect on liver regeneration [61, 71]. In addition to being secreted by HSCs, TGF-β is also secreted by KCs [72]. In short, KCs cannot be regarded as a single factor that hinders regeneration. In fact, during the regeneration process, macrophages are multifunctional cells [73, 74] (Fig. 2). A deeper understanding of this concept will also be the focus of our discussion.

#### Hepatocytes and BECs: bile as a pivotal mediator

Cholangiocytes, one of the main epithelial cells that constitute the liver parenchyma, are highly specialized cells that line the intrahepatic and extrahepatic bile ducts, participate in the production and homeostasis of bile [75]. Although the mechanism of interaction between hepatocytes and BECs in the PHx context is not fully understood, bile may be a pivotal mediator. Hepatocytes produce most of the bile, and BECs determine the final bile composition through a series of hormone- and neuropeptide-regulated secretion and absorption processes [76]. During injury and the resulting regeneration, the remnant liver faces a large potential overload of bile acids (BA), which exerts hepatoprotective effects through nuclear (mainly Farnesoid X receptor, FXR) and membrane (mainly G protein-coupled BA receptor 1, TGR5) receptors. On the one hand, hepatocyte cell cycle progression is also mainly promoted by the activation of the FXR-dependent transcription factor FoxM1b. On the other hand, TGR5 may be involved in the fine-tuning of cytokine production and release after PH, in a balanced manner that both protects the liver cells and promotes their growth factor-dependent progression into the cell cycle [77]. Vice versa, studies have shown that Yes-associated protein (YAP) signaling can promote the proliferation of BECs during regeneration after BA induction [78]. The above studies are of great significance for guiding clinical work, from a clinical point of view, extrabiliary drainage in human patients has been reported to be detrimental to the regenerating liver [79].

#### Communication between hepatocytes: autocrine cells and exosomes

In mammals, hepatocytes account for more than 80% of the liver mass and are one of the most effective cell types for maintaining homeostasis [1, 80]. During the regeneration process, hepatocytes not only crosstalk with nonhepatocytes but also communicate with each other by secreting cytokines or exosomes.

Transforming growth factor-alpha (TGF-α) is a growth factor secreted by hepatocytes that acts on hepatocytes [81]. It is a medium for communication among hepatocytes and is a ligand of epidermal growth factor receptor (EGFR). TGF-α is produced by hepatocytes and has 30% homology with EGF, which may trigger paracrine effects on hepatocyte stimulation, thereby promoting the proliferation of neighboring hepatocytes [1]. Notably, the elimination or knockdown of the TGF-α gene did not significantly affect liver regeneration. This may be because the receptor for TGF-α is EGFR, and there are many ligands for EGFR, including EGF, amphiregulin (AR), epiregulin (EREG), and heparin-binding EGF (HB-EGF) [82, 83]. Since these ligands bind to the same receptor, they may have complementary effects with each other, thereby offsetting the effects of TGF-α knockdown. Acidic fibroblast growth factor (aFGF), also known as HB-EGF, can act on hepatocytes in an autocrine manner to promote mitosis. The specific mechanism may be that aFGF reduces the inhibitory effect of TGF-β on DNA synthesis [84, 85].

In addition, hepatocytes can also secrete exosomes to regulate the proliferation of target hepatocytes. Exosomes can be secreted by a variety of cells, including hepatocytes, KCs, and endothelial cells [86–88]. Some researchers have found that exosomes derived from hepatocytes can induce hepatocyte proliferation, and the results are consistent in vivo and in vitro. In contrast, this phenomenon is not observed in response to exosomes derived from KCs or endothelial cells [89]. Sphingosine kinase 2 (SK2), which is unique in hepatocyte exosomes, is delivered to target hepatocytes and can induce the proliferation of target hepatocytes by inducing the synthesis of intracellular sphingosine-1-phosphate (S1P) [89]. This finding suggests that exosomes can be used as a means of communication between cells and play a role in liver repair and regeneration (Fig. 2).

### Crosstalk between nonhepatocytes cannot be ignored

#### HSCs and LSECs: indispensable to each other

HSCs and LSECs are closely connected anatomically, so they are closely related in function. HSC activation can regulate changes in the structure of endothelial cells and HSC proliferation. This process includes the recruitment of HSCs to endothelial cells and the secretion of angiogenic factors by HSCs to attract endothelial cells [90, 91].

During the revascularization process of liver regeneration, there are many signaling pathways that may be involved in mediating the recruitment of HSCs to blood vessels, and platelet-derived growth factors (PDGF) may be the most critical growth factor [91, 92]. PDGF is expressed by sprouting endothelial cells and binds to the PDGF receptor on HSCs, thereby inducing HSCs to accumulate near endothelial cells. In addition, TGF-β, angiopoietins, and NO are all involved in the recruitment of HSCs by endothelial cells [91].

After being recruited and activated by LSECs, HSCs in turn secrete factors to promote vascular remodeling during regeneration. The most important factor that HSCs first secrete and that acts on LSECs is VEGF. In addition to hepatocytes, HSCs are also an important source of VEGF. VEGF can promote the proliferation of endothelial cells, which indicates that crosstalk between LSECs and HSCs can promote the remodeling of blood vessels during regeneration (Fig. 2).

Additionally, from a structural and functional point of view, a single stellate cell wraps 4 sinuses and then controls sinus blood flow during regeneration by controlling the sinus [93]. This finding shows that the interaction between LSECs and HSCs has a potential regulatory effect on liver regeneration.

#### KCs and HSCs: bilateral regulation

Both KCs and HSCs are important nonhepatocytes in the liver. Interestingly, HSCs were first described and studied by Kupffer in 1876 [94]. The interaction between KCs and HSCs requires more in-depth research and has practical clinical significance.

HSCs secrete many factors that regulate cell proliferation and division and participate in all aspects of regeneration, including initiation, maintenance and termination [33, 95, 96]. Furthermore, there have been reports in the literature that HSCs are involved in ECM remodeling during regeneration [33]. This remodeling is very important for liver regeneration and can maintain the three-dimensional structure of regenerated cells [57]. KCs are essential for the recruitment of HSCs and the subsequent repair of the damaged liver [97]. HSCs are the main source of matrix metalloproteinases (MMPs) and their inhibitors [57, 98]. These cells participate in the regulation of ECM components, such as collagen, proteoglycans, glycosaminoglycans and glycoproteins, to produce temporary scars and prevent further damage [33]. Studies have shown that the mechanism by which HSCs regulate matrix remodeling involves inflammatory cytokines released by KCs [99]. The combined use of TNF-α and IL-1a in HSCs can enhance the expression of MMP1 and α-smooth muscle actin, which may be important regulators of tissue regeneration [99].

Notably, the interaction between KCs and HSCs is not unidirectional. HSCs also have regulatory effects on the recruitment and activation of immune cells during regeneration [100, 101]. Monocyte chemotactic peptide (MCP-1), which is secreted by HSCs, can stimulate KC infiltration [100, 102]. These recruited KCs or other immune cells not only have regulatory effects on liver regeneration but also provide additional signals, such as IL-13, to enhance the fibroblast activity of stellate cells to protect the liver [100] (Fig. 2).

#### HSCs and BECs: mesenchymal–epithelial interaction

In normal and regenerated livers, stellate cells exist in the progenitor cell niche near the Canals of Hering and are in close contact with BECs [103, 104]. The paracrine interactions between HSCs and BECs continues into adulthood, and the conditioned medium of adult HSCs promotes the growth of BEC lines to verify this view [105]. Therefore, it is reasonable to explore the mutual communication and interaction between HSCs and BECs during liver regeneration.

It has been demonstrated that BECs can attract lobular HSCs into portal tracts, and PDGF-BB is a key factor regulating this chemotaxis. Bile duct segments isolated from cholestatic rats increased the migratory capacity of HSCs, and this stimulation was significantly more effective than that of normal bile ducts. This suggests that BECs can attract the migration of HSCs in the context of cholestatic liver injury [105, 106]. Subsequently, the researchers further found that PDGF-BB released from tubular cells during bile duct injury promoted the activation and proliferation of HSCs. Therefore, early HSCs proliferation may be considered as an important defense mechanism aimed at alleviating liver damage and promoting liver regeneration [105, 107].

The flip side of things is how mesenchymal–epithelial interactions. Co-culture of HSCs and BECs showed that HSCs could produce Hedgehog (Hh) ligands, which enhanced the viability and proliferation of BECs [105]. The Hh signaling pathway leads to the activation of its downstream transactivators, including transcription factors of the Gli family, which regulate Hh target genes [105, 108].

#### LSECs and BECs: regulation of peripheral blood vessels

After liver parenchymal reduction, mature BECs can proliferate to restore the structure of the biliary tree, thereby regulating the evolution of liver injury. Proliferating BECs communicate and interact with other cells by secreting mediators that stimulate and activate multiple cell subtypes. BECs secrete vasoactive substances that regulate the remodeling of blood vessels supplying bile ducts to maintain desired nutritional and functional requirements [109]. When VEGF was blocked with a specific neutralizing antibody, peribiliary vascular plexus (PBP) proliferation did not occur after bile duct ligation (BDL), suggesting that VEGF secreted by BECs drives the proliferative adaptive response of PBP to cholestasis [110]. Another vasoactive substance secreted by proliferating BECs is endothelin-1 (ET-1), which is involved in the regulation of vascular bed function and plays an epicenter role in experimental hepatopulmonary syndrome after BDL [111].

#### KCs and BECs: the immunophysiology of biliary epithelium

During liver injury and the resulting regeneration, BECs interacted with inflammatory cells in vivo, suggesting that the immune system plays a pivotal role in liver regeneration. BECs secrete and transport immunoglobulins and produce cytokines and chemokines (IL-1, IL-6, IL-8, IFN-γ), recruit Kupffer cells to the portal vein [109]. The interaction mechanism between BECs and KCs is still not fully investigated, but the role of the immune system on BECs during liver regeneration will be the direction of future research.

#### KCs and LSECs: tightly connected

KCs reside in hepatic sinusoidal blood vessels, which are composed of LSECs, and attach to the surface of LSECs. The crosstalk between these two types of cells during regeneration influences the recruitment of KCs and the activation of LSECs [112]. Although the mechanism has not been fully explored, studies have shown that KCs are essential for the activation of LSECs and can make LSEC capillaries.

## Cellular crosstalk in transdifferentiation of hepatic epithelial cells

**Fig. 3:**
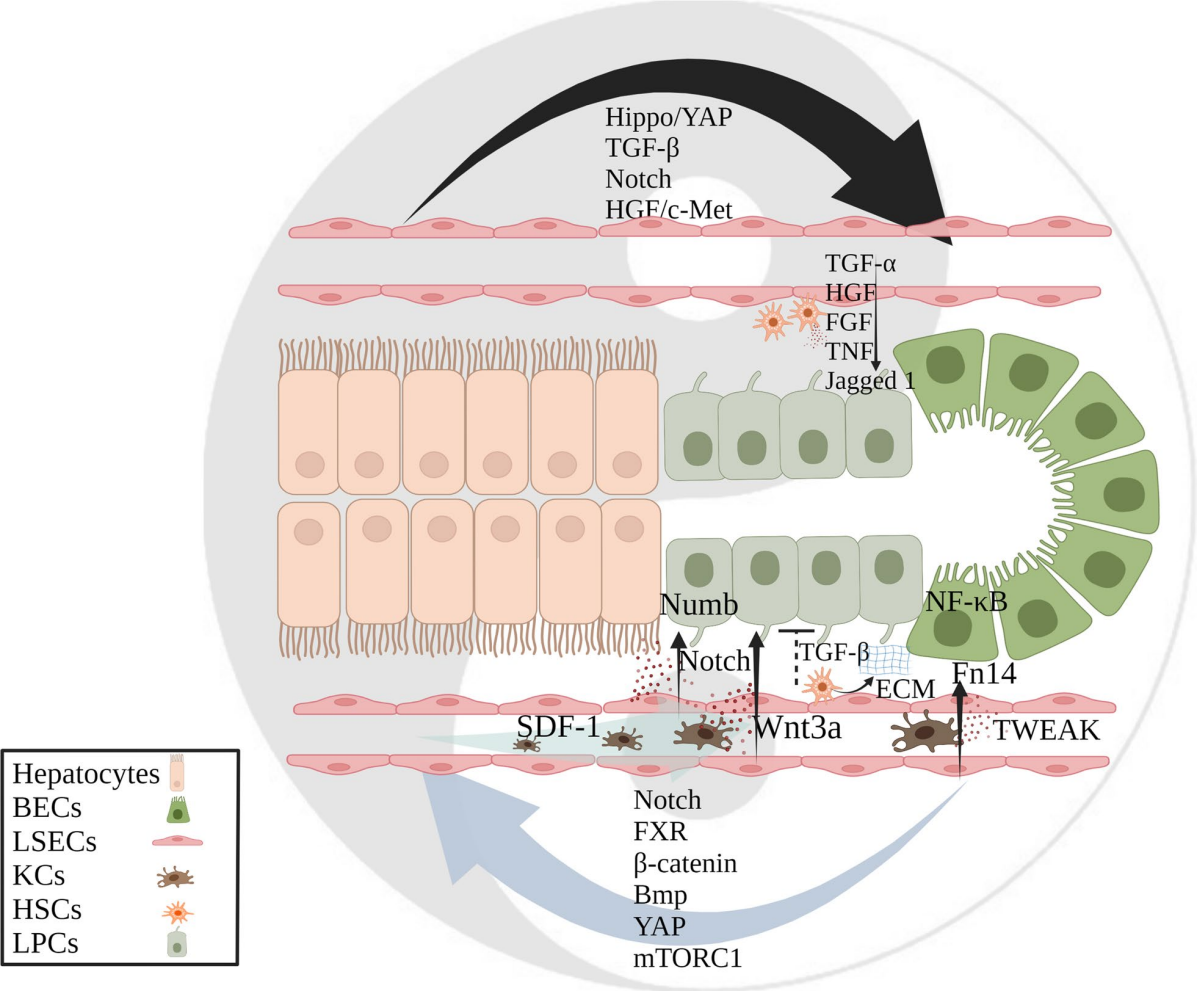
Cellular crosstalk in transdifferentiation of hepatic epithelial cells. Mechanisms required for reciprocal transdifferentiation of hepatocytes and cholangiocytes partially overlap; LSECs promote the transdifferentiation of LPCs by secreting cytokines; HSCs provide a specific microenvironment for the transdifferentiation of bipotential BECs by tightly cooperating with the ECM and cytokines/growth factors; KCs chemotactic by BECs can promote the transdifferentiation of liver epithelial cells by secreting cytokines

During liver regeneration, alternative regenerative mechanisms can occur once proliferation of resident epithelial cells is impaired. As shown in the ancient Chinese Taiji map, hepatic epithelial cells function as facultative stem cells and transdifferentiate into each other to restore normal liver structure (Fig. 3). There are still many unanswered questions surrounding the transformation of bipotential BECs into hepatocytes. Subsequently, we will discuss how various cells crosstalk with bi-potent BECs in the context of bipotential BECs -mediated liver regeneration.

### Hepatocytes and BECs: alternative regenerative pathways

Unlike the epidermis or blood, which have stem cells that constantly differentiate and replenish dead cells, hepatocytes and BECs can repair the lost liver through their own proliferation and division [113]. However, when the proliferation of a certain cell is damaged, additional cell expansion is needed [114, 115]. BECs or hepatocytes have specific markers. For example, cytokeratin 7 (CK7) or cytokeratin 19 (CK19) have been used to identify BECs, and hepatocyte nuclear factor 4 alpha (HNF4α) is also commonly used to label hepatocytes [114].

Transdifferentiation is a complete and stable change in cell identity that acts as an alternative to stem cell-mediated organ regeneration. By reducing the proliferation of hepatocytes during liver injury, the contribution of nonhepatocytes to parenchymal regeneration can be assessed. He et al. showed that almost all BECs in zebrafish (an animal model for studying liver regeneration) stably lost their tubular morphology, proliferate and expressed hepatocyte-specific markers after the extreme loss of hepatocytes, and this process requires transduction of Notch signaling, which activates SOX9b transcriptional factor in cholangiocytes [116]. In addition, FXR, β-catenin, bone morphogenetic protein (Bmp), YAP and mechanistic target of rapamycin complex 1 (mTORC1) [117–120] signaling all participate in BECs supplementation when hepatocyte proliferation is weakened. These results confirmed that once hepatocyte proliferation is inhibited, labeled BECs are directly involved in hepatocyte regeneration (Fig. 3).

In addition to biliary cell-driven liver regeneration, hepatocyte-driven biliary epithelial regeneration is also being studied. After preconditioning induced BEC damage, lineage tracking showed that the BECs were DPPIV + , suggesting that hepatocytes transdifferentiated into BECs during severe bile duct damage [121, 122]. BDL-induced injury of rat BECs showed that OV6/CK19 and SOX9/CK19 were increased and that the expression levels of Notch receptors and their ligands were also increased, suggesting that the Notch pathway plays an important role in this process [123]. Further research found that Notch-dependent hepatocyte-to-cholangiocyte reprogramming is regulated by signaling such as Hippo/YAP and TGF-β [124–126]. There is some overlap in the mechanisms of the two types of transdifferentiation, but this is not contradictory. Because both directions of transdifferentiation involve dedifferentiation of epithelial cells to form bipotential intermediates, Notch may be a key signaling pathway [116]. In addition, the HGF/c-Met pathway may also be related to the transdifferentiation of hepatocytes into BECs [127]. This can be evidenced by the inhibition of PI3-K (the downstream target of HGF/c-Met signal transduction) (Fig. 3).

Although the mechanism of BEC regeneration is not fully elucidated, it is critical to clinical patients. It is well known that in end-stage chronic liver disease, the ability of hepatocytes to proliferate is lost. If the compensatory ability of BECs could be stimulated, it would provide a new treatment strategy for clinical patients. Of course, this strategy also faces many challenges. For example, it is not clear how to more safely stimulate the regeneration potential of BECs or whether regeneration through transdifferentiation can rebuild the structure of the liver. These are the obstacles we will encounter in the future.

### LSECs and bipotential BECs: promote transdifferentiation of bipotential BECs

LSECs interact not only with blood components and form a sinusoidal barrier [93] but also with other cells in the liver to play a regulatory role in regeneration. In the early stage of regeneration, hepatocytes and BECs self-renew to form avascular cell clusters, which then stimulate the proliferation of LSECs to form blood vessels to transport nutrients.

The specific mechanism of the bidirectional regulation between LSECs and BECs during liver regeneration is still not fully understood. Studies have shown that LSECs can regulate Notch signaling, which is essential for biliary tract differentiation and bile duct formation [128, 129]. This finding suggests that LSECs are closely related to BECs during regeneration.

In addition, LSECs are also believed to interact closely with the Canals of Hering, which are located at the end of BECs. Studies have combined mouse liver progenitor cells (LPCs) with mouse LSECs to generate hepatobiliary organs with a liver-specific vascular system [130]. These studies show improved differentiation of hepatobiliary tissue and survival after transplantation (Fig. 3).

### HSCs and bipotential BECs: activation of bipotential BECs

In addition to their roles in the context of the self-replication of hepatocytes and cholangiocytes described above, HSCs are closely anatomically and physiologically linked to the intralobular tubule system and biliary tree, and can secrete a variety of growth factors, including TGF-α, HGF, FGF and TNF, which are required for bipotential BECs growth and proliferation [131, 132]. The collection of conditional medium (CM) from HSCs to 2-acetylaminofluorene in conjunction with PHx oval cell proliferation model demonstrated that HSCs promoted bipotential BECs DNA synthesis by paracrine HGF in the early stage of liver regeneration, and exerted an antagonistic effect through TGF-β1 in the later stage of liver regeneration [132]. Moreover, aHSCs can express Jagged 1 to promote Notch signaling in bipotential BECs, thereby promoting biliary specificity to their BECs [133].

Furthermore, aHSCs are involved in ECM remodeling during regeneration, which is tightly coupled with cytokines/growth factors to provide a specific microenvironment for bipotential BECs migration and anchoring [131]. Infiltration of liver parenchyma by bipotential BECs chaperoned by α-SMA-positive cells suggests that HSCs may be the major cellular source of ECM required for bipotential BECs proliferation and lobular invasion [134] (Fig. 3).

### KCs and bipotential BECs: the biliary tract and the immune system

Macrophages, which are represented by KCs, play a complex and contradictory role in the regeneration process, which may be related to the heterogeneity of macrophages and different stages of regeneration [74, 112]. BECs are also an important epithelial cell in the liver. The ability of BECs to supplement hepatocyte proliferation has been described in detail [114–116].

Regarding the localization of KCs in the biliary tract, C-X-C chemokine receptor 4 (CXCR4^+^) cells are recruited to the biliary tract via SDF-1 [135]. Duct-localized hepatic macrophages express TWEAK, whereas BECs express Fn14, which has been shown to mediate duct proliferation through NF-κB activation. This suggests that the TWEAK/Fn14 signaling pathway is a key component of macrophage-stimulated ductular reactions (DR) activation [136].

During the process of liver regeneration, bipotential BECs, which are typically wrapped in a thick layer of myofibroblasts and type I collagen, are exposed and come into contact with macrophages [133, 137, 138]. Subsequently, KCs phagocytosis of hepatocyte debris induces Wnt3a expression, which leads to the canonical Wnt signaling in bipotential BECs, thereby maintaining Numb expression (a cell fate determinant) within these cells and promoting their specification to hepatocytes [133, 139]. Here, we summarize the mechanism by which bipotential BECs are able to respond to diverse cellular microenvironments for divergent cell fates (Fig. 3).

### Bipotential BECs and surrounding cells: awaiting exploration

Compared with studies on the effect of surrounding cells on the transdifferentiation of bipotential BECs, there are still unanswered questions about the mechanism of crosstalk between surrounding cells in the context of transdifferentiation. Studies have shown that the interaction between HSCs and KCs plays a regulatory role in the differentiation of LPCs [133]. In the future, the interactions among other cells are still waiting for researchers to explore.

## Conclusions

Although the liver rarely undergoes regeneration under physiological conditions, under pathological conditions, such as hepatectomy and liver failure, the liver can restore its volume and function through its powerful regenerative capacity. The process of liver regeneration is attributed to the interaction of both independent and interrelated cells in the liver. Hepatocytes are in a multicellular environment, and the effects of other cells can change their regenerative capacity. Moreover, crosstalk exists among nonhepatocytes in the liver. There have been few studies on this aspect of the interaction, and this is one of the highlights of the present article.

In the present review, we mainly described the intrahepatic crosstalk network during liver regeneration. Indeed, the liver is not an isolated organ, and studies have reported that extrahepatic organs also have a regulatory effect on the regenerated liver, including the thyroid, adrenal gland, pancreas, duodenum, and autonomic nervous system [69]. Moreover, the infiltration of immune cells (especially T lymphocytes and NK cells) plays an important role in regulating regeneration [140–142], such as preventing liver damage and removing damaged cells. The interaction between the symbiotic bacteria in the intestine and the liver can also regulate liver regeneration [143]. These results demonstrated that further understanding of the regulation of liver regeneration by the extrahepatic system is also a promising direction, which will be our focus in a future study.

The exact mechanism underlying liver regeneration is complicated and still not well defined. However, with emerging technologies such as genetic lineage tracking, we believe a comprehensive mechanism will be illustrated, which will contribute to the recovery of hepatectomy patients and improve therapeutic options among patients with liver failure.

## Data Availability

Not applicable.

## Abbreviations

NPCs: Nonparenchymal cells
LSECs: Liver sinusoidal endothelial cells
PHx: Partial hepatectomy
ALPPS: Associating liver partition and portal vein ligation for staged hepatectomy
HSCs: Hepatic stellate cells
KCs: Kupffer cells
BECs: Biliary epithelial cells
BM-SPCs: Bone marrow-derived sinusoidal epithelial progenitor cells
SDF-1: Stromal cell-derived factor 1
CXCR7: CXC chemokine receptor 7
VEGF: Vascular endothelial growth factor
HGF: Hepatocyte growth factor
KLF2: Kruppel-like factor 2
SFSS: "Small-for-size" syndrome
PVF: Portal vein flow
MCS: Mesocaval shunt
FEDA: Fibronectin extra domain A
HIF: Hypoxia inducible factor
ECM: Extracellular matrix
u-PA: Urokinase plasminogen activator
MAPK: Mitogen-activated protein kinase
ROS: Reactive oxygen species
NP: Norepinephrine
TGF-β: Transforming growth factor-beta
EGF: Epidermal growth factor
5-HT2B: 5-Hydroxytryptamine receptor subtype 2B
HNF4α: Hepatocyte nuclear factor 4 alpha
BMP: Bone morphogenetic protein
YAP: Yes-associated protein
mTORC1: Mechanistic target of rapamycin complex 1
BDL: Bile duct ligation
TNF-α: Tumor necrosis factor-α
IL-6: Interleukin 6
LPS: Lipopolysaccharide
Jak: Janus kinase
SH2: Src homology 2
Bcl-2: B-cell lymphoma-2
EGFR: Epidermal growth factor receptor
AR: Amphiregulin
EREG: Epiregulin
HB-EGF: Heparin-binding EGF
aFGF: Acidic fibroblast growth factor
SK2: Sphingosine kinase 2
S1P: Sphingosine-1-phosphate
PDGF: Platelet-derived growth factors
MMPs: Matrix metalloproteinases
MCP-1: Monocyte chemotactic peptide
LPCs: Liver progenitor cells
TWEAK: TNF-related weak apoptosis inducers
BA: Bile acids
YAP: Yes-associated protein
PBP: Peribiliary vascular plexus
ET-1: Endothelin-1
CM: Conditional medium
DR: Ductular reactions
